# Efficacy and safety of different fractions in stereotactic body radiotherapy for spinal metastases: A systematic review

**DOI:** 10.1002/cam4.2546

**Published:** 2019-09-05

**Authors:** Yining Gong, Lingyi Xu, Hongqing Zhuang, Liang Jiang, Feng Wei, Zhongjun Liu, Yan Li, Miao Yu, Kaiwen Ni, Xiaoguang Liu

**Affiliations:** ^1^ Department of Orthopedics Peking University Third Hospital Beijing China; ^2^ Eight‐Year‐Program, Grade 2015 Health Science Center Peking University Beijing China; ^3^ Department of Radiation Oncology Peking University Third Hospital Beijing China; ^4^ Research Center of Clinical Epidemiology Peking University Third Hospital Beijing China

**Keywords:** multi‐fraction, single‐fraction, spinal metastases, stereotactic body radiotherapy, stereotactic radiosurgery, systematic review

## Abstract

**Background:**

In the treatment of spinal metastases, stereotactic body radiotherapy (SBRT) delivers precise, high‐dose radiation to the target region while sparing the spinal cord. A range of doses and fractions had been reported; however, the optimal prescribed scheme remains unclear.

**Methods:**

Two reviewers performed independent literature searches of the PubMed, EMBASE, Cochrane Database, and Web of Science databases. Articles were divided into one to five fractions groups. The Methodological Index for Non‐randomized Studies (MINORS) was used to assess the quality of studies. Local control (LC) and overall survival (OS) were presented for the included studies and a pooled value was calculated by the weighted average.

**Results:**

The 38 included studies comprised 3,754 patients with 4,731 lesions. The average 1‐year LCs for the one to five fractions were 92.7%, 84.6%, 86.8%, 82.6%, and 80.6%, respectively. The average 1‐year OS for the one to five fractions were 53.0%, 70.4%, 60.1%, 48%, and 80%, respectively. The 24 Gy/single fraction scheme had a higher 1‐year LC (98.1%) than those of 24 Gy/two fractions (85.4%), 27 Gy/three fractions (84.9%), and 24 Gy/three fractions (89.0%). The incidence of vertebral compression fracture was 10.3%, with 10.7% in the single‐fraction group and 10.1% in the multi‐fraction group. The incidence of radiation‐induced myelopathy was 0.19%; three and two patients were treated with single‐fraction and multi‐fraction SBRT, respectively. The incidence of radiculopathy was 0.30% and all but one patient were treated with multi‐fraction SBRT.

**Conclusions:**

SBRT provided satisfactory efficacy and acceptable safety for spinal metastases. Single‐fraction SBRT demonstrated a higher local control rate than those of the other factions, especially the 24 Gy dose. The risk of vertebral compression fracture (VCF) was slightly higher in single‐fraction SBRT and more patients developed radiculopathy after multi‐fraction SBRT.

## Graphical abstract

Single‐fraction SBRT demonstrated a higher local control than other factions, especially with 24Gy dose. The risk of VCF was slightly higher in single‐fraction SBRT and more patients developed radiculopathy after multi‐fraction SBRT.

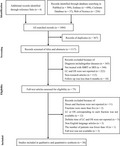

## 1 INTRODUCTION

Bone metastases are the most common tumor metastases after lung and liver metastases; among bone metastases, the spine is the most common site.^1^, ^2^ About 40% of patients with cancer develop spinal metastases.^3^ With advances in systemic therapy, patient survival has improved significantly and patients benefit more from improved local control (LC).^4^ Conventional external beam radiotherapy (cEBRT) had been the principal therapy for spinal metastases and was effective for symptom palliation with improved LC and overall survival (OS).^5^, ^6^ However, the low‐dose tolerance of critical adjacent organs at risk (OARs) made the desired dose unachievable and this low‐dose radiotherapy was not optimized for prognosis, especially in radioresistant histology types.^7^ Therefore, stereotactic body radiotherapy (SBRT) or stereotactic radiosurgery (SRS) has emerged for use in spinal metastases. Compared to cEBRT, this method provides relatively better pain relief and LC. In one multicenter and matched‐pair study,^8^ the perioperative visual analogue scale (VAS) score decrease was larger in the SRS group and progression‐free survival differed significantly between the two groups.

SBRT is a highly conformal radiotherapy that delivers precise, high‐dose radiation to target regions while sparing the spinal cord.^9^ With the widespread adaption of SBRT, studies have demonstrated its efficacy and safety.^10^, ^11^, ^12^ Even in radioresistant tumor types, SBRT offers an effective treatment with favorable LC.^11^ The prescribed scheme may be an important predictor for LC.^13^ A range of total doses and fraction numbers have been reported for SBRT and several studies have compared different fraction schemes, with the role of single‐fraction SBRT supported by some studies,^14^, ^15^ while others reported no significant differences,^16^ or that multi‐fraction SBRT was superior.^17^ The preferred dose and fraction pattern of SBRT for the treatment of spinal metastasis remained unknown. Therefore, we performed this systematic review to identify the efficacy and toxicity of different fractions in SBRT for spinal metastases.

## 2 METHODS

### 2.1 Literature search

This systematic review was conducted in accordance with the guidelines of Preferred Reporting Items for Systematic Reviews and Meta‐Analyses (PRISMA)^18^ and was registered in PROSPERO (CRD42019120479). Two reviewers performed independent literature searches of the PubMed, EMBASE, Cochrane Database, and Web of Science databases. The search strategy is shown in Table S1. The date of the last search was February 1, 2019. The reference lists of the identified articles and reviews were manually screened for additional eligible studies.

### 2.2 Study selection

Title/abstract and full‐text reviews were carried out successively and separately by two reviewers. Studies that satisfied the following criteria were selected. The inclusion criteria were: (a) articles with a confirmed diagnosis of spinal metastases; (b) patients treated with SBRT or SRS; (c) reported LC and/or OS; (d) a minimum of 6‐month follow‐up. The exclusion criteria were: (a) cohorts including the diagnosis of other diseases; (b) fewer than 10 patients; (c) unreported doses or fractions; (d) more than five fractions; (e) missing information on detailed LC and/or OS corresponding to each fraction or median/mean fraction; (f) missing information on the definite time of LC and OS; (g) non‐English language articles; (h) nonclinical research articles; and (i) full text not available.

### 2.3 Data collection

A specialized database was established by searching for the first author, publication time, study design, demographic characteristics, histology of the primary tumor, radiological scheme (dose and fraction), and patient outcomes (eg, local control and overall survival). After initial review, the articles were divided according to the number of fractions (one to five). Three approaches were used to determine the fraction group to which the studies belonged. First, all patients in the study were treated with the same fraction. Second, the median fraction was reported for the patients. Third, more than 75% of patients were treated with the same fraction. Data extraction was also performed independently by two reviewers.

### 2.4 Assessment of quality of the evidence

The quality of the evidence was assessed with specific scores according to the Methodological Index for Non‐randomized Studies (MINORS).^19^ The established protocol and prospective database were both regarded as a prospective collection of data. The appropriate follow‐up time was defined as no less than 1 year. The global score was 16 for noncomparative studies and 24 for comparative studies. All assessments were performed independently by two reviewers, with differences resolved by discussion to reach a consensus.

### 2.5 Data analysis

LC and OS were determined for the included studies and the pooled values were calculated by the weighted average. Data were presented as frequencies and percentages. The number of patients/lesions was estimated from the number of lesions/patients if not available. A meta‐analysis was not performed due to the lack of studies with comparative design as well as the heterogeneity of interventions.

## 3 RESULTS

**Figure 1:**
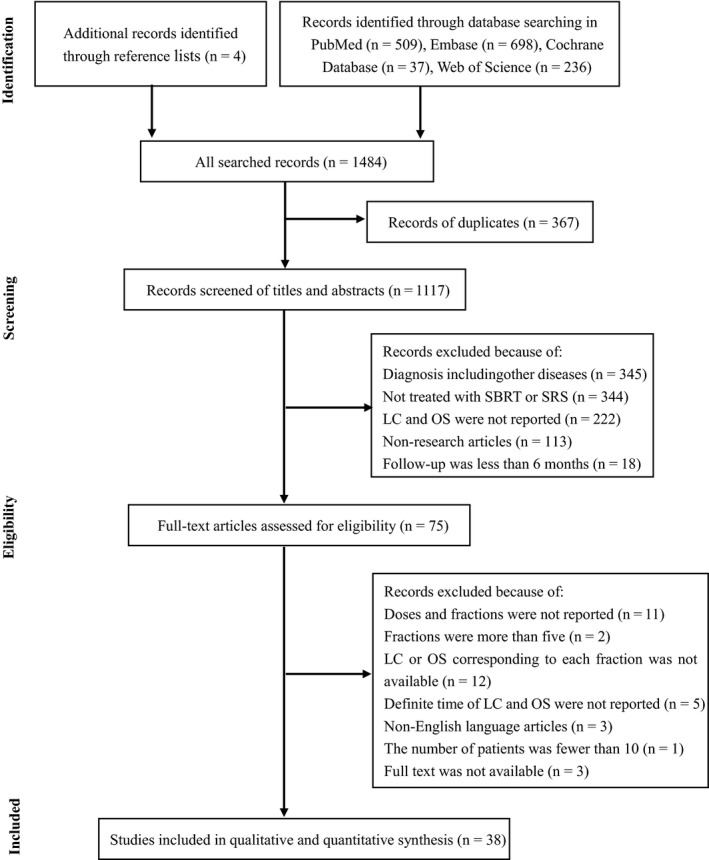
Flow of information through the different phases of the systematic review. SBRT, stereotactic body radiotherapy; SRS, stereotactic radiosurgery; LC, local control; OS, overall survival

A total of 1480 studies were identified initially and another four eligible studies were added following reference screening. After removing duplicates, screening the titles and abstracts, and assessing full texts, 38 studies were included in the analysis. The flow of information through the different phases is shown in Figure 1. No randomized controlled trial (RCT) was identified. Five studies were prospective and the rest were retrospective designs. The publication years ranged from 2009 to 2019. This review included 3754 patients with 4731 lesions. The MINORS scores and study characteristics are presented in Tables 1, 2, 3, 4, 5.

**Table 1:** Studies using single‐fraction SBRT for spinal metastases

Reference	Patients (Lesions)	Age (median)	Sex (male/female)	Tumor type	Dose (Gy, median)	Follow‐up (mo, median)	Local control (1‐y)	Overall survival (1‐y)	MINORS
Yamada et al^11^	657 (811)	NA	NA	mixed	24	26.9	99.4%	NA	14
Virk et al^12^	323 (552)	60.7	201/121	mixed	24	12.6	NA	58.8%	12
Kumar et al^16^	20 (NA)	NA	NA	mixed	24	20	95%	NA	19
Ghia et al^15^	NA (21)	NA	NA	RCC	24	23	95%	NA	22
Folkert et al^14^	NA (68)	NA	NA	sarcoma	24	12.3	90.8%	70.7%	20
Laufer et al^20^	40 (NA)	NA	NA	mixed	24	7.6	91%	NA	12
Moulding et al^21^	21 (NA)	53.2	15/6	mixed	24	10.3	90.5%	NA	12
Garg et al^22^	61 (63)	60	34/27	mixed	16‐24	19.7 (mean)	88% (18‐mo)	64% (18‐mo)	14
Bate et al^23^	NA (38)	NA	NA	mixed	16‐23	10 (overall)	97.4%	NA	20
Hashmi et al^24^	215 (247)	62	104/111	mixed	18	8.1	83%	48%	12
Nikolajek et al^25^	54 (70)	56	32/22	mixed	18	14.5	88%	NA	12
Heron et al^17^	NA (195)	59	63/61	mixed	16.3 (mean)	12	70% (2‐y)	46%	20
Miller et al^26^	38 (56)	59	22/16	MM	16	26	91%	NA	14
Miller et al^27^	249 (NA)	60.6	155/125	mixed	16	18 and 12^a^	81.9%	NA	20
Amdur et al^28^	21 (25)	NA	NA	mixed	15	8	95%	25%	14

Abbreviations: Gy: gray; MM: multiple myeloma; mo: month; NA: not applied; RCC: renal cell carcinoma; y: year.

a Median follow‐up for instrumentation cohort and control cohort;

**Table 2:** Studies Using Two‐Fraction SBRT for Spinal Metastases

References	Patients (Lesions)	Age (median)	Sex (male/female)	Tumor type	Dose (Gy, median)	Follow‐up (mo, median)	Local control (1‐y)	Overall survival (1‐y)	MINORS
Zeng et al^29^	52 (93)	NA	27/25	mixed	24	14.4 and 19.5^a^	91.3%	61.5%	22
Ito et al^30^	131 (134)	65	81/50	mixed	24	9	72.3%	65.0%	12
Ito et al^31^	28 (28)	62	18/10	mixed	24	13	70%	NA	12
Tseng et al^32^	145 (279)	68	78/67	mixed	24	15	90.3%	73.1%	14
Chang et al^33^	60 (72)	66	49/11	mixed	24	21	92%	90%	12
Thibault et al^34^	37 (71)	63	25/12	RCC	24	12.3	83%	64%	14
Choi et al^35^	42 (51)	57	17/25	mixed	20	7	73%	68%	12
Tsai et al^36^	69 (127)	54	34/35	mixed	15.5 (mean)	10	96.8% (10‐mo)	NA	11

Abbreviations: Gy: gray; mo: month; NA: not applied; RCC: renal cell carcinoma; y: year.

a Median follow‐up for cervical cohort and sacral cohort respectively;

**Table 3:** Studies using three‐fraction SBRT for spinal metastases

Reference	Patients (Lesions)	Age (median)	Sex (male/female)	Tumor type	Dose (Gy, median)	Follow‐up (mo, median)	Local control (1‐y)	Overall survival (1‐y)	MINORS
Folkert et al^14^	NA (52)	NA	NA	Mixed	28.5	12.3	84.1%	46.2%	20
Wang et al^37^	149 (166)	58	77/72	Mixed	27‐30	15.9	80.5%^a^	71.9%	15
Silva et al^38^	NA (20)	NA	NA	Mixed	27	13.58	87.8%	NA	12
Ghia et al^15^	NA (26)	NA	NA	RCC	27	23 (overall)	71%	NA	22
Park et al^39^	39 (59)	61	24/15	Mixed	27	7.4	93.2%	47.4%	12
Garg et al^40^	59 (63)	60	35/24	Mixed	27	17.6 (mean)	76%	76%	14
Laufer et al^20^	37 (NA)	NA	NA	Mixed	27	7.6	95.9%	NA	12
Mehta et al^41^	83 (98)	64	47/36	Mixed	24	7.6	84%	46%	12
Anand et al^42^	52 (76)	58	30/22	Mixed	24	8.48	94%	68%	12
Guckenberger et al^43^	301 (387)	61.3	166/135	Mixed	24	11.8	89.9%	64.9%	12
Kim et al^44^	22 (31)	56	9/13	Mixed	24	10	81.3% (6‐mo)^a^	64.5% (6‐mo)	11
Ahmed et al^45^	66 (85)	56.8	48/18	Mixed	24	8.2	89.2%	52.2%	12
Sahgal et al^46^	39 (60)	NA	NA	Mixed	24	9 and 7^b^	85%	45% (2‐y)	20
Puvanesarajah et al^47^	99 (NA)	60.4	51/48	Mixed	21	6.1	NA	43.7%	12

Abbreviations: Gy, gray; mo, month; NA, not applied; RCC, renal cell carcinoma; y, year.

a Tumor progression‐free survival rates.

b Median follow‐up for unirradiated and reirradiated group, respectively.

**Table 4:** Studies using four‐fraction SBRT for spinal metastases

References	Patients (Lesions)	Age (median)	Sex (male/female)	Tumor type	Dose (Gy, median)	Follow‐up (mo, median)	Local control (1‐y)	Overall survival (1‐y)	MINORS
Thibault et al^48^	40 (56)	58	25/15	mixed	30	6.8	81%	48%	14
Sohn et al^8^	13 (NA)	62.1	NA	RCC	38^a^	NA	85.7%	NA	20

Abbreviations: Gy, gray; mo, month; NA, not applied; RCC, renal cell carcinoma; y, year.

a Mean total margin radiation dose.

**Table 5:** Studies using five‐fraction SBRT for spinal metastases

References	Patients (Lesions)	Age (median)	Sex (male/female)	Tumor type	Dose (Gy, median)	Follow‐up (mo, median)	Local control (1‐y)	Overall survival (1‐y)	MINORS
Silva et al^38^	NA (52)	NA	NA	mixed	35	13.5	81.2%	NA	12
Gill et al^49^	20 (NA)	NA	6/14	mixed	30	34	80%	80%	12

Abbreviations: Gy, gray; mo, month; NA, not applied; y, year.

### 3.1 Efficacy

#### 3.1.1 Single‐fraction group

Through assessment and discussion, 15 studies^11^, ^12^, ^14^, ^15^, ^16^, ^17^, ^20^, ^21^, ^22^, ^23^, ^24^, ^25^, ^26^, ^27^, ^28^ were categorized into the single‐fraction group (Table 1). A total of 2021 patients with 2476 lesions were included. The median follow‐up time of all included studies ranged from 7.6 to 26.9 months. The average LC and OS at 1 year (doses ranging from 15 to 24 Gy) were 92.7% (1545 of 1666 lesions) and 53.0% (436 of 822 patients), respectively. Garg et al reported LC and OS of 88% and 64%, respectively, at 1.5 years.^22^ The LC was 70% at 2 years with a single‐fraction scheme and the mean dose was 16.3 Gy.^17^ The most common single‐fraction dose was 24 Gy. In patients administered 24 Gy in a single fraction, the average 1‐year LC and OS were 98.1% (962 of 981 lesions) and 60.9% (238 of 391 patients), respectively.

#### 3.1.2 Two‐fraction group

Eight studies included two‐fraction groups (Table 2).^29^, ^30^, ^31^, ^32^, ^33^, ^34^, ^35^, ^36^ A total of 564 patients with 855 lesions were analyzed. The median follow‐up time of the included studies ranged from 7 to 21 months. The average 1‐year LC and OS (doses ranging from 15.5 to 24 Gy) were 84.6% (616 of 728 lesions) and 70.4% (329 of 467 patients), respectively. Six studies administered schemes delivering 24 Gy in two fractions. The average 1‐year LC and OS were 85.4% (578 of 677 lesions) and 70.8% (301 of 425 patients), respectively, in patients administered 24 Gy in two fractions. However, the LC at 10 months was as high as 96.8% in the study by Tsai et al, with a mean dose of 15.5 Gy.

#### 3.1.3 Three‐fraction group

This review included 14 studies with three‐fraction schemes (Table 3).^14^, ^15^, ^20^, ^37^, ^38^, ^39^, ^40^, ^41^, ^42^, ^43^, ^44^, ^45^, ^46^, ^47^ There were 1044 total patients and 1259 lesions. The median follow‐up time in the included studies ranged from 6.1 to 23 months. The average 1‐year LC and OS (doses ranging from 21 to 30 Gy) were 86.8% (980 of 1129 lesions) and 60.1% (541 of 900 patients), respectively. Median doses of 27 and 24 Gy were delivered in five and six studies, respectively. The average 1‐year LC and OS for a median dose of 27 Gy were 84.9% (174 of 205 patients) and 64.3% (63 of 98 patients), respectively. Kim et al reported 6‐month LC and OS for a median dose of 24 Gy of 81.3% and 64.5%, respectively.^44^ The average LC and OS at 1 year in other studies with doses of 24 Gy were 89.0% (628 of 706 lesions) and 60.4% (303 of 502 patients), respectively. Sahgal et al reported a 2‐year OS of 45%.^46^

#### 3.1.4 Four‐fraction group

This review included only two studies with four‐fraction schemes (Table 4).^8^, ^48^ In total, 53 patients with 69 lesions were analyzed. The prescribed doses in the two studies were 30 and 38 Gy, respectively. The median follow‐up time was 6.8 months and the 1‐year OS was 48% (19 of 40 patients) in the study by Thibault et al.^48^ The average 1‐year LC was 82.6% (57 of 69 lesions).

#### 3.1.5 Five‐fraction group

Two studies reported the outcome of five‐fraction schemes (Table 5).^38^, ^49^ Seventy‐two patients with 72 lesions were included. The prescribed doses were 35 and 30 Gy, respectively. The median follow‐up time for the two studies were 13.5 and 34 months, respectively. The average 1‐year LC was 80.6% (58 of 72 lesions). The 1‐year OS was 80% in the study by Gill et al^49^

### 3.2 Safety

#### 3.2.1 Vertebral compression fractures (VCF)

Twenty studies reported the occurrence of VCF after SBRT for spinal metastases (Table 6).^8^, ^12^, ^15^, ^22^, ^23^, ^24^, ^26^, ^27^, ^29^, ^30^, ^31^, ^32^, ^33^, ^34^, ^38^, ^39^, ^41^, ^43^, ^44^, ^47^ The single‐fraction and multi‐fraction arms in the studies by Ghia et al and Bate et al are listed separately in Table 6.^15^, ^23^ A total of 2686 lesions in 2074 patients were included. The median follow‐up time of all included studies ranged from 6.1 to 26 months. The average incidence of VCF after SBRT was 10.3% (278 of 2686 lesions). Six studies reported the time to VCF, which ranged from 1.2 to 15.4 months. The average incidence of VCF after SBRT in the single‐fraction (doses ranging from 16 to 24 Gy) and multi‐fraction (doses ranging from 20 to 38 Gy) groups were 10.7% (130 of 1218 lesions) and 10.1% (148 of 1468 lesions), respectively.

**Table 6:** Studies reported VCF after SBRT for spinal metastases

References	Patients (Lesions)	Tumor type	Dose (Gy)/Fraction	Follow‐up (mo, median)	VCF	Time to VCF (mo)
Virk et al^12^	323 (552)	mixed	24/1	12.6	7.2% (5‐y)	13
Ghia et al^15^	NA (13)	mixed	24/1	23	46.2%	NA
Garg et al^22^	61 (63)	mixed	16‐24/1	19.7 (mean)	21.3%	NA
Bate et al^23^	NA (38)	mixed	16‐23/1	10	2.6%	NA
Hashmi et al^24^	215 (247)	mixed	18/1	8.1	4.5%	NA
Miller et al^26^	38 (56)	MM	16/1	26	21%	NA
Miller et al^27^	249 (NA)	mixed	16/1	18 and 12^a^	18.9%	NA
Zeng et al^29^	52 (93)	mixed	24/2	14.4 and 19.5^b^	3.8%	NA
Tseng et al^32^	145 (279)	mixed	24/2	15	13.8% (2‐y)	NA
Ito et al^30^	131 (134)	mixed	24/2	9	11.9%	NA
Ito et al^31^	28 (28)	mixed	24/2	13	10.7%	NA
Chang et al^33^	60 (72)	mixed	24/2	21	6.7%	15.4
Thibault et al^34^	NA (61)	RCC	24/2	12.3	16%	NA
Bate et al^23^	NA (31)	mixed	20‐30/2‐5	10	12.9%	NA
Ghia et al^15^	NA (11)	mixed	27/3 and 30/5	23	9.1%	NA
Silva et al^38^	61 (72)	mixed	27/3 and 35/5	13.58	20.8%	NA
Park et al^39^	39 (59)	mixed	27/3	7.4	5.1%	1.2 and 1.4^c^
Guckenberger et al^43^	301 (387)	mixed	24/3	11.8	7.8%	11.5
Mehta et al^41^	83 (98)	mixed	24/3	7.6	4.2%	5.8
Kim et al^44^	22 (31)	mixed	24/3	10	19.4%	NA
Puvanesarajah et al^47^	99 (NA)	mixed	21/3	6.1	7.0%	3.3
Sohn et al^8^	13 (NA)	RCC	38^d^/4	NA	15.4%	NA

Abbreviations: Gy: gray; MM: multiple myeloma; mo: month; NA: not applied; VCF: vertebral compression fractures; y: year.

a Median follow‐up for instrumentation cohort and control cohort.

b Median follow‐up for cervical cohort and sacral cohort.

c Time to VCF for two patients.

d Mean total margin radiation dose.

#### 3.2.2 Radiation‐induced myelopathy and radiculopathy

The occurrence of radiation‐induced myelopathy or radiculopathy was rarely reported. Twenty‐two studies reported whether there were complications of radiation myelopathy or radiculopathy.^11^, ^14^, ^15^, ^23^, ^24^, ^27^, ^29^, ^30^, ^31^, ^32^, ^33^, ^35^, ^37^, ^38^, ^39^, ^40^, ^41^, ^43^, ^44^, ^46^, ^47^, ^48^ Only five and eight patients developed radiation‐induced myelopathy^11^, ^27^, ^31^, ^35^ and radiculopathy,^15^, ^29^, ^30^, ^40^, ^41^ respectively. Pooling of the patients from these 22 studies showed an incidence of radiation‐induced myelopathy of 0.19% (5 of 2659 patients), and an incidence of radiculopathy of 0.30% (8 of 2659 patients). Three of the five patients who developed radiation‐induced myelopathy were treated with single‐fraction SBRT while the other two were treated with multi‐fraction SBRT. All but one of the patients who developed radiculopathy was treated with multi‐fraction SBRT.

#### 3.2.3 Other toxicities

Other toxicities of SBRT for spinal metastases are shown in Table S2. Fatigue, dysphagia, pain flare, dermatitis, and esophagitis were common toxicities. The occurrence of grade 3 and grade 4 toxicities was rare. Ghia et al reported no difference in the rates of toxicity between single‐fraction and multi‐fraction groups.^15^

## 4 DISCUSSION

Different fractions may provide different outcomes and toxicities.^14^, ^15^, ^50^ However, the utilization of single‐ and multi‐fraction SBRT remains controversial. We conducted this systematic review with a large sample size to evaluate the efficacy and safety of different prescribed schemes to help identify an optimal dose and fraction pattern for SBRT for spinal metastases.

This systematic review considered only one to five fractions since they were more frequently adopted and fraction numbers more than five would likely decrease the efficacy of SBRT.^14^, ^51^ The results supported the efficacy and safety of SBRT in this population. The average LC and OS at one year were 88.9% (3256 of 3664 lesions) and 59.6% (1341 of 2249 patients) for all fractions. In addition, the safety was acceptable based on the incidence of VCF, radiation‐induced myelopathy, and radiculopathy.

The average 1‐year LC for each fraction was 92.7%, 84.6%, 86.8%, 82.6%, and 80.6%, respectively. Satisfactory LC was observed in all fractions but appeared to be more effective in the single‐fraction scheme. This finding was consistent with those of other reports indicating that a single fraction was correlated with significantly improved LC compared to that of multi‐fractions.^14^, ^15^ Folkert et al reported a superior 1‐year LC in the single‐fraction group (90.8% vs 84.1%, *P* = .007) and remained significant in multivariate analysis.^14^ The LC was still higher in the single‐fraction group at two years (86% vs 55%, *P* = .009) in the study by Ghia et al.^15^ At the 3‐year follow‐up, the LC rates were 84% and 56% in the single‐fraction and multi‐fraction cohorts, respectively, in the study by Kumar et al, but the difference was not statistically significant (*P* = .20).^16^ However, one study reported the opposite results. Heron et al showed a significantly better LC in the multi‐fraction group than that in the single‐fraction group (96% vs 70%, *P* = .001) up to 2 years posttreatment and the need for retreatment was significantly lower in the multi‐fraction group (1% vs 13%, *P* < .001).^17^

In terms of OS, the average 1‐year OS for each fraction were 53.0%, 70.4%, 60.1%, 48%, and 80%, respectively. OS was highest in the five‐fraction group and lowest in the four‐fraction group. Only one study reported OS both in four‐fraction and five‐fraction groups. In the remaining three schemes, the 1‐year OS in the single‐fraction group was lower than that in the other two groups. Similar results were observed by Heron et al, in which the 1‐year OS was significantly higher in the multi‐fraction group than that in the single‐fraction group (63% vs 46%, *P* = .002).^17^ Other studies reported no difference between single‐fraction and multi‐fraction groups.^14^, ^15^

The delivered dose is reported to be a significant factor for LC.^11^, ^13^ Thus, different doses in the same fraction may predict different outcomes. We selected studies using specific common dose schemes in each fraction for subgroup analysis, such as 24 Gy in a single fraction, 24 Gy in two fractions, and 27 or 24 Gy in three fractions.^52^ Our results were similar to those previously reported. The 24 Gy/single fraction scheme had a higher 1‐year LC than those of the 24 Gy/two fractions, 27 Gy/three fractions, and 24 Gy/three fractions schemes. However, the 1‐year OS was lower than those of the 24 Gy/two fractions and 27 Gy/three fractions schemes.

The safety of SBRT for spinal metastases was evaluated by VCF, radiation‐induced myelopathy, radiculopathy, and other toxicities. VCF occurred in about one‐tenth of patients after SBRT in our study. The frequencies of symptomatic VCF requiring intervention following single‐fraction SBRT were 0.31%, 1.9%, 2.2%, 2.9%, 5.4%, 6.6%, and 7.2% at 3 months, 6 months, 1 year, 2 years, 3 years, 4 years, and 5 years, respectively.^12^ Thibault et al reported that the incidence of VCF was higher in the single‐fraction group than that in the multi‐fraction group (25% vs 9%, *P* = .028). However, the incidence was almost equal in our study (10.7% vs. 10.1%). Few studies reported the time to VCF, which ranged widely (1.2‐15.4 months). Therefore, patients may have been at risk for VCF for a long time after SBRT. Although the incidence of radiation‐induced myelopathy and radiculopathy were low, each patient requires careful management. Among the patients who developed radiation‐induced myelopathy, similar numbers received single‐fraction and multi‐fraction SBRT; however, more patients treated with multi‐fraction SBRT developed radiculopathy.

Our study had several limitations. No RCT and only a few studies with prospective designs were included. The patients in each fraction were mixed and we did not perform subgroup analyses such as types of tumor histology and radiation (de novo, reirradiation, and postoperative SBRT) because some studies did not provide data on these specific classifications. The patients were treated with different fractions and could not be separated in some studies. Therefore, the median fraction was used to classify these studies. In addition, four studies in which more than 75% of patients were treated with three fractions were assigned to the three‐fraction group, which may have introduced some bias. Comparison studies were limited and there was heterogeneity in interventions; thus, a meta‐analysis could not be performed to directly and precisely compare efficacy and safety between different fractions.

In conclusion, SBRT provided satisfactory efficacy and acceptable safety for spinal metastases. Single‐fraction SBRT demonstrated a higher LC than those of other fractions, especially the 24 Gy dose. The risk of VCF was slightly higher in single‐fraction SBRT and more patients developed radiculopathy after multi‐fraction SBRT. More studies with higher levels of evidence and comparative designs are needed to confirm these findings.

## CONFLICT OF INTEREST

We declare that there is no conflict of interest to this work.

## Data Availability

I confirm that my article contains a Data Availability Statement even if no data are available (list of sample statements) unless my article type does not require one. I confirm that I have included a citation for available data in my references section, unless my article type is exempt.
