# Supplementary material for: Efficacy and safety of different fractions in stereotactic body radiotherapy for spinal metastases: A systematic review
Source: Cancer Med. 2019 Sep 5;8(14):6176–84. doi: 10.1002/cam4.2546 (PMC6797563; doi:10.1002/cam4.2546)
Supplement: Supplementary file 1 [file CAM4-8-6176-s001.docx]

Supplemental Table 1. The Developed Search Strategy Performed in Database of PubMed

| No | Search terms |
| --- | --- |
| #1 | Stereotactic radiosurgery |
| #2 | SRS |
| #3 | #1 OR #2 |
| #4 | Stereotactic body radiotherapy |
| #5 | SBRT |
| #6 | #4 OR #5 |
| #7 | Radiosurgery |
| #8 | #3 OR #6 OR #7 |
| #9 | Spin* metastases |
| #10 | Spinal metastatic disease |
| #11 | Metastatic spinal tumor |
| #12 | Spinal bone metastases |
| #13 | #9 OR #10 OR #11 OR #12 |
| #14 | #8 AND #13 |

Supplemental Table 2. Studies Reported Other Toxicities after SBRT for Spinal Metastases

| Reference | Patients (Lesions) | Dose (Gy)/Fraction | Toxicities (n=patients) |
| --- | --- | --- | --- |
| Silva et al. 2019^38^ | 61 (72) | 27/3 and 35/5 | Grade 1: fatigue (n=8), upper gastrointestinal mucosal (n=6), and skin (n=2);  Grade 2: fatigue (n=1); |
| Miller et al. 2017^28^ | 249 (NA) | 16/1 | Grade 3: esophagitis (n=1);  Unclear: pain flare (n=32); |
| Chang et al. 2017^33^ | 60 (72) | 24/2 | Acute  Grade 1: including fatigue, nausea, pain flare, and oesophagitis (n=10);  Grade 2: transient radiculitis and diarrhoea (n=3);  Late  Grade 2: VCF causing pain (n=3);  Grade 3: VCF requiring a stabilisation procedure (n=1); |
| Hashmi et al. 2016^24^ | 215 (247) | 18/1 | Unclear: dysphagia (n=24), dermatitis (n=6), and pain flare (n=27) (mild to moderate in 18 of these cases and severe in 9); |
| Ghia et al. 2016^15^ | 43 (47) | 24/1, 27/3 and 30/5 | Unclear: pain flare (n=13); |
| Anand et al. 2015^42^ | 52 (76) | 24/3 | Unclear: pain flare (n=2); |
| Sohn et al. 2014^8^ | 13 (NA) | 38^†^/4 | Unclear: nausea, abdominal discomfort, and dysphagia, et al. (n=5); |
| Guckenberger et al. 2014^43^ | 301 (387) | 24/3 | Grade 1: dermatitis (n=11), dysphagia (n=27), and pain (n=29);  Grade 2: dysphagia (n=2) and pain (n=18);  Grade 3: pain (n=2); |
| Folkert et al. 2014^14^ | 88 (120) | 24/1 | Acute  Grade 1: dermatitis (n=48), fatigue (n=19), esophagitis (n=14 patients), nausea (n=3 patients), and musculoskeletal pain (flare) (n=2);  Grade 2: fatigue (n=7), esophagitis (n=4), and dermatitis (n=3);  Grade 3: dermatitis (n=1);  Chronic  Grade 1: fatigue (n=13), neuropathy (n=7), dermatitis/fibrosis (n=5), musculoskeletal pain (n=3), gastrointestinal (cramping/reflux) (n=2), insufficiency fracture (n=2), and shortness of breath (n=1);  Grade 2: fatigue (n=2) and esophagitis (n=1);  Grade 3: tracheoesophageal fistula (n=2), fatigue (n=1), and wound complication (n=1); |
| Kim et al. 2013^44^ | 22 (31) | 24/3 | Grade 2: esophagitis (n=2); |
| Wang et al. 2012^37^ | 149 (166) | 27-30/3 | Grade 1&2: transient numbness and tingling, nausea, and vomiting (n=NA);  Grade 3: nausea (n=1), vomiting (n=1), diarrhea (n=1), fatigue (n=1), non-cardiac chest pain (n=3), dysphagia (n=1), neck pain (n=1), diaphoresis (n=1), and pain associated with severe tongue edema and trismus (n=2); |
| Gill et al. 2012^49^ | 20 (NA) | 30/5 | Grade 1&2: acute dysphagia/esophagitis (n = 4); |
| Garg et al. 2012^22^ | 61 (63) | 16-24/1 | Grade 1: neurotoxicity (n=6), gastrointestinal (n=9), musculoskeletal (n=7), and other toxicity (n=9);  Grade 2: neurotoxicity (n=3), gastrointestinal (n=3), musculoskeletal (n=6), and other toxicity (n=5);  Grade 3: neurotoxicity (n=1);  Grade 4: neurotoxicity (n=1); |
| Ahmed et al. 2012^45^ | 66 (85) | 24/3 | Grade 1 (n=12);  Grade 2 (n=6);  Grade 3 (n=2); |
| Garg et al. 2011^40^ | 59 (63) | 27/3 | Grade 1: neurotoxicity (n=7), gastrointestinal (n=6), and other toxicity (n=19);  Grade 2: neurotoxicity (n=4), gastrointestinal (n=6), and other toxicity (n=16);  Grade 3: neurotoxicity (n=2); |
| Moulding et al. 2010^21^ | 21 (NA) | 24/1 | Grade 1: skin (n=3);  Grade 2: esophagitis (n=3);  Grade 4: esophagitis (n=1); |
| Tsai et al. 2009^36^ | 69 (127) | 15.5/2 | Grade 1&2: fatigue (n=50%), nausea (n=27%), vomiting (n=16%), esophagitis (n=11%), diarrhea (n=3%), sore throat (n=5%), anemia (n=1%), thrombocytopenia (n=2%), and neutropenia (n=4%); |
| Sahgal et al. 2009^46^ | 39 (60) | 24/3 | Grade 1&2: (n=3); |
| Bate et al. 2015^23^ | 57 (69) | 16-23/1 and 20-30/2-5 | Unclear: mild esophagitis (n=1); |
| ^†^ Mean total margin radiation dose;  Gy: gray; NA: not applied. | | | |
